# The “Trojan Horse” Approach to Tumor Immunotherapy: Targeting the Tumor Microenvironment

**DOI:** 10.1155/2014/789069

**Published:** 2014-05-18

**Authors:** Delia Nelson, Scott Fisher, Bruce Robinson

**Affiliations:** ^1^School of Biomedical Sciences, CHIRI Biosciences Research Precinct, Curtin University, Perth, WA 6102, Australia; ^2^National Centre for Asbestos Related Diseases, UWA School of Medicine and Pharmacology, Harry Perkins Institute of Medical Research, QEII Medical Centre, Perth, WA, Australia

## Abstract

Most anticancer therapies including immunotherapies are given systemically; yet therapies given directly into tumors may be more effective, particularly those that overcome natural suppressive factors in the tumor microenvironment. The “Trojan Horse” approach of intratumoural delivery aims to promote immune-mediated destruction by inducing microenvironmental changes within the tumour at the same time as avoiding the systemic toxicity that is often associated with more “full frontal” treatments such as transfer of large numbers of laboratory-expanded tumor-specific cytotoxic T lymphocytes or large intravenous doses of cytokine. Numerous studies have demonstrated that intratumoural therapy has the capacity to minimizing local suppression, inducing sufficient “dangerous” tumor cell death to cross-prime strong immune responses, and rending tumor blood vessels amenable to immune cell traffic to induce effector cell changes in secondary lymphoid organs. However, the key to its success is the design of a sound rational approach based on evidence. There is compelling preclinical data for local immunotherapy approaches in tumor immunology. This review summarises how immune events within a tumour can be modified by local approaches, how this can affect systemic antitumor immunity such that distal sites are attacked, and what approaches have been proven most successful so far in animals and patients.

## 1. Introduction

Most anticancer therapies, including immunotherapies, are given systemically but little attention has been given to therapies given directly into tumors. There is a powerful logic for such an approach—after all, the most profound tissue destructive immune processes are “driven” by local factors which overcome the natural suppressive/protective factors in the tissue environment, suppressive/protective factors that are used by tumors to escape destruction. There is compelling preclinical data for local immunotherapy approaches in tumor immunology and we will summarise these data in this paper.

**Figure 1:**
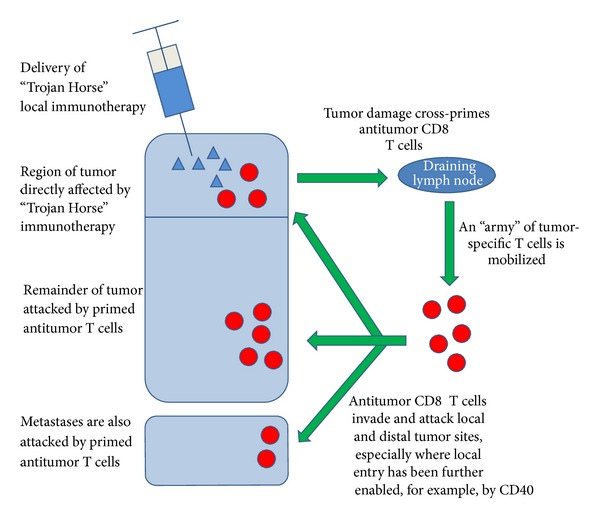
Conceptual illustration of the “Trojan horse” approach to tumor immunotherapy. An immune modulator is delivered directly into a portion of the tumor. That results in inflammation plus “dangerous death”. This results in mobilization of an “army” of tumor specific T cells which then attack the area of the tumor injected* plus* uninjected areas of tumor, especially if addition agents are provided which promote access of these T cells into these areas and/or local stimulation, for example, agonistic anti-CD40 antibodies.

It is important to understand that this approach seeks not only to induce destruction of the tumor site injected but to also induce a more widespread response which then destroys uninjected local and metastatic tumor deposits. We call this the “Trojan Horse” approach because, in the ancient Greek tale, a full front “systemic” approach against a walled city was not successful, even after a 10-year siege, so they penetrated the host defences by subterfuge, using a wooden horse in which soldiers were hidden. Once inside, the small number of soldiers were sufficient to overcome local defences and open the gate to allow the main Greek force to enter and destroy the city; that is, the main force was then able to mobilise and defeat the enemy. Local immunotherapy of cancer aims to do much the same thing. This concept is illustrated in Figure 1.

Targeting reagents directly into the tumor microenvironment to induce tumor regression is not a new concept. Paul Ehrlich dreamt of a “magic bullet” that could be used to target diseased tissues and organs. Whilst Ehrlich predicted that the immune system could repress the growth of carcinomas and it was William B. Coley who demonstrated that activating the immune systems in patients using heat killed bacterial cultures from* Streptococci *and* Serratia marcescens* could induce tumor regression. Coley tried multiple regimens with his concoction including comparing intratumoural (i.t.) versus intravenous (i.v.) administration (reviewed by [1, 2]). His studies suggested that only patients who developed a strong local and systemic inflammatory response, measured by increased body temperature, tumour necrosis, and tumor edema, were likely to benefit. Importantly, the closer to the tumor the injections were given, the better the outcome is, implying a role for the draining lymph nodes and thus priming for a systemic response—more about that later. In Coley's days the complexities of the immune system and the tumor microenvironment were barely understood. A large volume of work has now shown that manipulating the tumor microenvironment by local or distal means using reagents that directly (e.g., cytokines) or indirectly (e.g., cytotoxic reagents) activate components of the immune systems can induce tumor regression and provide a permanent cure. Nonetheless, whilst there are proof-of-principle studies showing the power of the anticancer immune response, we still do not have a robust treatment approach that can reliably treat most patients with different cancers at different stages of disease.

We reason [3] that effective antitumor immune responses require a similar profound and complex response to that seen in antipathogen responses, as implied by Coley. Responses to infection represent coordinated local and systemic immune responses. Activation of tissue-resident cells induces blood vessels to become amenable to the trafficking of large numbers of innate and adaptive immune cells into, and out of, the site of infection. Pathogen-associated antigens sourced from the infected site are transported to draining lymph nodes where long-term effector/memory T and B cells immunity is generated. Similarly, we propose that strategies that modulate key compartments of the tumor microenvironment via direct tumor-targeting approaches combined with strategies the drive effector T and B cells in draining lymph nodes will be most effective.

## 2. The Trojan Horse Approach

Our own laboratory studies, discussed in this review, led us to the development of the “Trojan Horse” concept which then led to several clinical trials. Rather than commencing therapy by using a full frontal assault to attack tumors (such as adoptive therapy of large numbers of laboratory expanded tumor-specific CTLs or large i.v. doses of cytokine), we chose instead to first deliver reagents directly into tumors aiming to induce microenvironmental changes at the same time as avoiding systemic toxicity. This involved minimising local suppression and/or inducing sufficient “dangerous” tumor cell death to cross-prime strong immune responses and/or rending tumor blood vessels amenable to immune cell traffic to induce effector cell changes in secondary lymphoid organs. There is a large body of work demonstrating the safety and feasibility of directly injecting solid tumors, and new strategies are being developed. It is hoped that this approach will be more effective and less toxic for a broad range of cancers at different stages of disease; our data suggest this is possible. However, the key to its success is the design of a sound rational approach based on evidence.

## 3. Targeting the Tumor Microenvironment

In order to modify any tumor to induce immune-mediated damage, the nature of the tumor environment and the factors preventing such damage first need to be understood. The complexity of tumor microenvironments is becoming increasingly clear and interactions between the multitude of different immune and nonimmune cell types and heterogenetic tumor cells that occupy this space are not yet well understood. This environment consists of tumor and companion cells, the latter collectively described as stromal cells. Stromal cells include cells that make up tumor-associated blood vessels in particular endothelial cells and pericytes; cells that contribute to structural integrity (fibroblasts); as well as tumor-associated macrophages (TAMs) and infiltrating immune cells including monocytes, neutrophils (PMN), dendritic cells (DCs), T and B cells, mast cells, and natural killer (NK) cells. Indeed, stromal cells make up the bulk of tumor cellularity and in many cases the dominating cell type in solid tumors is the macrophage [4–6]. Thus, tumor cells may represent a minority cell type within a tumor making them harder to target. This knowledge has led many researchers to reconsider how best to attack a solid tumor, and modifying stromal elements is becoming increasingly attractive.

Adding further complexity are data showing that solid tumors consist of different “microniches.” Some niches are well perfused and oxygenated, whilst others are poorly perfused and hypoxic. The latter niche is particularly dangerous to the host as it is inaccessible via systemic drug delivery and it harbours resistant tumor cells that can survive a nutrient and oxygen deprived environment [7]. Each niche is likely to represent its own mini-microenvironment with different dominating cell types and a different soluble factor milieu.

## 4. What the Cytokine Milieu Is Like in Tumors and How This Needs to Be Altered

The tumor cytokine milieu consists of tumor and immune cell-derived pro- and anti-inflammatory factors. Tumors may contain proinflammatory IL-6 [8–10] that contributes to angiogenesis and cancer cachexia, as well as transforming growth factor beta (TGF-*β*), a product of regulatory CD4^+^ T cells (Tregs) which suppress effector immune cell function and contribute to tumor escape from host immune surveillance [11]. Other proinflammatory factors found in tumors include TNF-*α* and IFN-*γ* ([5] and our unpublished data), which may be TAM or T cell derived; they may occupy a niche in which tumor cells are being killed, or they may contribute to a chronically inflamed niche that is protumorigenic. Anti-inflammatory mediators include vascular endothelial growth factor (VEGF) which drives angiogenesis [12] and disables DC function and migration [13, 14] and IL-10. Whilst there is clear evidence that IL-10 is immunosuppressive and protumorigenic [15, 16], there is also new contradictory evidence that IL-10 can enhance apoptosis and downregulate VEGF, TNF-*α*, and IL6 production by TAMs to suppress angiogenesis and tumor growth [17]. These different IL-10-related effects may depend on interactions with other local factors [18]. It is likely that the combination and concentration (or domination) of specific cytokines dictate the outcome of a cancer niche. For example, a combination of TNF-*α*, IL-6, and IL-17 may compromise antitumor immunity and, if in sufficient levels throughout the tumor bed, promote tumor progression. In contrast, a combination of TRAIL, IL-10, and IL-12 can lead to tumor suppression [18]. Thus, perturbing the tumor cytokine milieu may lead to important beneficial downstream effects.

## 5. Evidence That Immune Selection Occurs in the Tumor Microenvironment

There is good evidence that even at early stages the tumor microenvironment can permit immune destruction under the right circumstances. It is now accepted that cancer immunosurveillance is a process in which the host immune system recognizes and eliminates early or quiescent tumor cells [19, 20]. However, when incomplete, cancer immunosurveillance is proposed to be the first step of a deadly process, termed cancer immunoediting, in which tumor antigen-specific T cells “sculpt” tumor cells to select those that are completely resistant to T cell killing [19]. Selection may occur initially at the site of the draining lymph node then at the tumor sites [21]. Thus, the tumor microenvironment is dynamic and constantly changing as the process of immunoediting continues through the last two phases, that is, equilibrium and escape. Equilibrium represents a period of immune-mediated latency. However, due to their genetic instability, tumor cells become less immunogenic (e.g., via tumor antigen loss, MHC class I downregulation, etc.) and easily avoid T cell attack leading to the final phase of escape. Escape is the potentially fatal outgrowth of immune-resistant tumor cells that invade other tissues [19].

## 6. Key Processes That Have to Be Modified in the Tumor Microenvironment

Features that have to be modified in the tumor microenvironment and hence allow effective antitumor responses include rendering tumor blood vessels more permissive to bidirectional immune cell traffic. Either tumor cells or tumor antigen-laden dendritic cells (DCs) need to be able to exit tumors to transport their antigenic load to draining lymph nodes [21, 22], whilst effector immune cells and antibodies need to be able to readily access tumor cells throughout the tumor bed. Suppressive elements within the tumor microenvironment need to be silenced or repolarised to promote local inflammation. In particular, attention must be paid to TAMs, myeloid derived suppressor cells (MDSCs), and regulatory T cells (Treg) that likely collaborate to induce potent local suppression [23].

## 7. Modulating Tumor-Associated Blood Vessels

In our studies, targeting agonist anti-CD40 antibody (Ab) into the tumor bed generated important local changes. In particular, tumor-associated blood vessels became permissive to T cell immigration [24] and DC emigration [25]. Transient normalisation of tumor vessels is reported to lead to better perfusion of the tumor bed by cytotoxic chemotherapy [26] and, likely, improved tumor penetration of antibodies and other important molecules. Conversely, targeting CD40 may also be protumorigenic. Chiodoni et al. demonstrated that anti-CD40 Ab could activate endothelial cells and enhance tumor neoangiogenesis in a murine model of mammary carcinoma [27]. Thus, while a single local change in tumor vessels could allow more widespread destruction of tumor cells and increased export of tumor antigen to draining lymph nodes, it may be a double edged sword. Therefore, a considered approach to the design of targeted CD40 therapy is needed to ensure that this more stealthy approach can pave the way for further immune modulation, using standard cytotoxic chemotherapy or immunotherapy.

## 8. Using the Tumor Site as Its Own Source of Antigen Stimulation of a Systemic Response

One attractive therapeutic option that we have explored is to modify the tumor microenvironment so that it acts as its own source of antigenic stimulation, or as a vaccine. In this case, cytotoxic drugs can be used to increase tumor cell killing. Alternatively, the numbers/activation/function of CTLs and/or NK cells could be increased to contribute to* in situ* tumor cell killing. Antigen presenting cell phagocytosis of live or dead tumor cells can be increased, particularly in the draining lymph nodes. One attraction of this approach is that the tumor antigens do not need to be identified—the death of the tumor cells delivers a dose of tumor antigens into the cross-priming pathway. We have shown that all of the above can be achieved in mesothelioma and lung cancer models either by vaccination with heat shocked autologous tumor [28] or intratumoral injection of IL-2 combined with anti-CD40 Ab, which avoids toxicity issues and induces regression of large primary tumors, as well as distal tumors [29].

**Figure 2:**
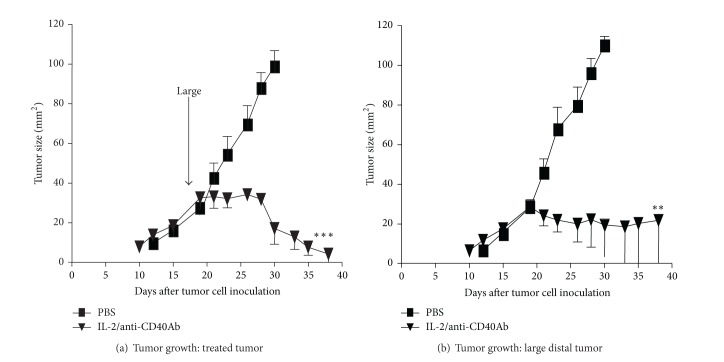
Animal study showing how intratumoral therapy induces local regression plus distal regression of uninjected tumor. Intratumoural IL-2 + anti-CD40 is an effective combination against local tumour and untreated distal tumours. C57Bl/6J mice were injected s.c. with 5 × 10^5^ AE17 tumour cells into the left flank and 5 × 10^5^ AE17 cell into the right flank on day 0 and left to develop into large tumours before treatment began. The i.t. treatments were delivered into one tumour indicated by arrows (treated tumour: a), whilst the second (distal) tumour was left untreated (b). Data from 1 experiment (5 or 6 mice/group) is shown as mean ± SEM. Treated mice were compared to PBS-treated controls mice; **P < 0.01, ***P < 0.001 (from Jackaman et al., 2008. International Immunology) [29].

The need to prove that such an approach can induce a systemic response which is active at distal sites and provides protective memory is essential to the notion of using a tumor as its own vaccine. However, few studies have systematically examined the impact of i.t. immunotherapy on local versus global (systemic) antitumor immunity and long-term memory. Local anti-CD40 Ab administration has been shown to eradicate treated-site tumors and induce a systemic CTL response that eradicates distal tumors [27, 30]. We have shown that generating local inflammation via IL2/anti-CD40 Ab therapy [29] results in collaborating CD4^+^ and CD8^+^ T cells that patrol the body to eradicate distal untreated tumors (Figure 2) and protect from rechallenge [31]. Interestingly, we found that different effector mechanisms can operate to eradicate treated site versus untreated distal tumors; that is, when agonist anti-CD40 Ab is combined with IL-2, the local effector response bypasses CD4^+^ help; however, collaborating CD4^+^ and CD8^+^ T cells were critically required for eradicating untreated distal tumors and for long-term protection [31]. We also identified a unique role for NK cells in this setting. NK cells did not function as effector cells after local IL-2/anti-CD40 Ab treatment. However, NK cells not only contributed to systemic immunity leading to the resolution of untreated distal tumors, but also provided help for the acquisition and maintenance of long-term effector/memory T cell responses [32]. These data suggest that treatment-dependent responses generated within tumors and draining lymph nodes determine whether tumor-specific, effector/memory T cells are disseminated to patrol the entire body seeking other tumors.

## 9. Why Cross-Presentation within Tumors Is Important

There is clear evidence that tumor-specific CTLs can directly recognise tumor cells. However, tumor antigen is not likely to be directly presented by tumor cells to CD8^+^ T cells in draining lymph nodes; therefore, cross-presentation may be the only form of natural antigen presentation for tumor immunity (reviewed in [33]). Thus, antigen presenting cells, in particular DCs that have acquired tumor antigen whilst in the tumor microenvironment, or from tumor cells killed in the draining lymph nodes [21] could be targeted. In this process, exogenous antigen derived from tumor cells is taken up by DCs and processed and displayed in MHC class I molecules for presentation to CD8^+^ T cells, rather than following the classical pathway of processing exogenous antigen for presentation in MHC class II molecules to CD4^+^ T cells. Importantly, cross-presenting DCs need to be appropriately activated for presentation to occur with the appropriate costimulation; otherwise, this process will result in weak T cell responses or tolerance (reviewed by [34, 35]). Whilst some of the key cross-presentation events occur in lymph nodes that drain the tumors, it is clear that DCs in tumors may also be important. Such DCs are often in an immature activation state and are devoid of costimulatory molecules. Indeed it has been shown that such DCs are blocked in their capacity to cross-present tumour antigens locally to “restimulate” CTLs [35]. Such DCs could promote local and regional Treg expansion [36]. DC maturation, function, and migration capacity can be suppressed by local MDSCs, M2 macrophages, and Tregs [37]. Thus, the tumor microenvironment must be deliberately modulated such that local suppression is alleviated and DCs can respond to activation signals cell that aid the cross-presentation process. It has recently been shown that the chemotherapy agent gemcitabine can reverse the block in intratumoral DC cross-presentation [35, 38].

## 10. Reducing the Immune Suppression That Is Present within Tumors

Even when there is local destruction of tumor cells and there is appropriate stimulation of the antigen presentation pathway, the resultant effector response does not always destroy tumors. This is because increasing tumor burden is associated with accumulation of a range of escape mechanisms. That means that no amount of “accelerator pressure” will work unless the “brakes” are released. These brakes include increasing numbers of suppressive immune cell types infiltrating the tumor microenvironment to further dampen CTL function. Thus, these cells also need to be targeted to be silenced or reprogrammed. Given their numerical domination in most solid tumors reprograming M2 TAMs into M1 macrophages may be particularly effective [30, 39]. This has been achieved using bacteria [40], microRNA, [41, 42] LPS/IFN*γ* [39], and IL-2/anti-CD40 Ab [43]. We propose that local therapy to reprogram M2 TAMs may be more effective and less toxic than systemic therapy allowing sufficient local destruction to occur to then prime a systemic host antitumor immune response.

MDSCs are immature myeloid cells induced by tumor-derived factors in tumor-bearing hosts. MDSCs are a complex and as yet poorly understood suppressive immune cell type that can be found in lymphoid organs and tumors in most cancer patients where they inhibit innate antitumor immunity [44, 45]. Interestingly, MDSCs have been shown to be superior to other immune cell types in preferentially migrating to tumors rather than other tissues. This observation prompted the design of an engineered strain of oncolytic vesicular stomatitis virus (VSV) that binds to MDSCs for their transport into the tumor microenvironment. Direct tumor killing was further accentuated by promoting MDSC differentiation towards the classically activated M1-like phenotype [46]. Thus, targeting MDSCs may remove another layer of immune suppression in tumors.

Similar to MDSCs, Tregs are able to infiltrate the tumour milieu and their presence is often associated with disease progression and poor prognosis [47, 48]. Developing tumours may produce an array of cytokines, chemokines, and other soluble factors that promote enrichment of Treg within the tumour microenvironment. CCR4 is highly expressed on Treg relative to conventional T cells and facilitates Treg migration toward tumours expressing the CCR4 ligands CCL17/CCL21 [49–51], while other studies have shown that Tregs expressing VEGF-A (CXCR4) are preferentially attracted to tumours expressing the angiogenic promoting chemokine CXCL12 [52, 53]. Other potential mechanisms of Treg enrichment within tumours include expression cytokines that (i) induce conversion of conventional T cells into Treg or (ii) preferentially expand a small number of tumour resident Treg over other cell types (reviewed in [54]).

The therapeutic efficacy of Treg targeted immunotherapies has been demonstrated in numerous preclinical studies in which targeted depletion of Tregs was observed to improve antitumour immunity and shown to prevent tumour development and decreased established tumour progression [55–57]. In the clinic, similar outcomes have been observed, albeit without complete tumour regression, with protocols aiming at reducing Treg using cyclophosphamide alone [58], or as combination therapies [59, 60]. These studies demonstrate the critical role of Treg in suppressing antitumour immunity and highlight the importance of gaining a complete understanding of their role during tumour development so that more effective immunotherapies can be developed.

## 11. Targeting T Cell Cosignalling to Enhance Local Effectiveness of Cancer Immunotherapy

Local therapy may also modify “costimulation.” The classic two-signal model of full T cell activation involving TCR recognition of antigen in the context of MHC (signal 1) coupled with binding of CD28 to B7.1/B7.2 (CD80/CD86; signal 2) has been expanded upon the number of cosignalling molecules being discovered increases (reviewed in [61]). Cosignalling receptors may modulate T cell signalling in either a positive (costimulatory) or negative (coinhibitory) manner and thus ultimately dictate the outcome of T cell activation. As such, there has been an increased focus on recent years on developing treatments modalities that specifically target these costimulatory and coinhibitory receptors as a means to enhance anticancer immunotherapies.

Cytotoxic T lymphocyte a*∖*Antigen 4 (CTLA-4), also known as CD152, is considered an important immune regulatory checkpoint that is expressed by T cells, in particular Tregs, and counteracts the costimulatory activity of CD28. CLTA-4 binds with higher affinity to CD80/CD86 and can directly outcompete binding of CD28 [62, 63] or when expressed by Tregs can act in a cell extrinsic manner to strip CD80/CD86 from the surface of activated APCs and thus significantly inhibit their ability to drive antitumor immunity [64]. Treatment with anti-CTLA-4 monoclonal antibody (mAb) is emerging as an anticancer therapy that blocks the inhibitory activity of CTLA-4 on effector T cells and Tregs resulting in enhanced antitumor effector T cell activity [65]. There is new evidence that anti-CTLA-4 mAb selectively depletes Tregs in tumor lesions and that this process is dependent on the presence of Fc gamma receptor-positive macrophages [66]. These data imply that targeting anti-CTLA-4 mAb into the tumor microenvironment may be particularly effective and avoid toxicity issues that have been reported in clinical trials [67].

Programmed cell death protein 1, also known as PD-1, is closely related to CTLA-4. PD-1 has two ligands, PD-L1 and PD-L2, which are members of the B7 family [68]. Similar to CTLA-4, PD-1 and its ligands negatively regulate T cell responses and monoclonal Abs targeting PD-1 have been developed to counteract immune regulation and boost antitumor immunity [69]. Responses to systemic treatment have been promising with complete or partial responses in non-small-cell lung cancer, melanoma, and renal-cell cancer, in a clinical trial with a total of 296 patients [70]. However, no responses were seen in colon and pancreatic cancer. These data highlight the fact that different cancers respond differently and that consistent effective regimens involving these immune checkpoint inhibitors have yet to be identified. There is also evidence that combination of anti-CTLA-4 Ab with anti-PD-1 Abs is more effective than use of either Ab on its own [71] and recent exciting studies have confirmed the effectiveness of this combination in human clinical trials [72]. It not yet clear whether targeting T cells in secondary lymphoid organs via systemic administration is the best way to move forward; however, we have evidence that anti-PD-1 Abs would be more effective in regional lymph nodes (our unpublished data). Additional studies have shown that both tumour and stromal cells can upregulate PD1 and its ligands in response to the local cytokine milieu (reviewed in [73]), limiting antitumour immunity. Thus, there is clear rational for targeting PD-1/-L1/-L2 immunotherapy directly into the tumour to produce more effective therapy.

The initial success of the CTLA-4 and PD-1 “checkpoint” immunotherapies motivated researchers to expand their investigations into targeting other known cosignalling receptors as potential agonistic immunotherapies. In particular, members of the tumor necrosis factor receptor super family (OX40, 4-1BB, and GITR) whose role as costimulatory receptors help maintain survival, effector function, and memory persistence of activated T cells [74]. Extensive preclinical studies indicate that agonistic OX40 therapy can promote antitumor immunity by simultaneously expanding effector T cells while blocking Treg mediated suppression [75–80], particularly when delivered intratumourally [79]. Similarly agonistic 4-1BB therapy may enhance tumor immunity by enhancing effector function and protecting it from programmed cell death [81, 82], while anti-GITR therapy has been associated with the ability to break tolerance to melanoma differentiation antigens [83] and augment Treg accumulation within tumors [84]. Importantly, the antitumor potential of agonistic OX40, 4-1BB, and GITR immunotherapies can be significantly enhanced when combined with conventional cancer treatments such as chemotherapy, radiotherapy, surgery, or other immunotherapies [77, 85–87].

The use of agonistic immunotherapies that target cosignalling molecules is still in its infancy. This niche field of cancer immunotherapy will continue to expand as our knowledge of cosignalling receptors (known and those possibly yet to be discovered) continues to increase. Their potential as immunotherapies will be dictated by our understating of the molecular interactions associated with their respective signalling pathways, their use as single agents or in combination with other immunotherapies, and ultimately their delivery method; local intratumoral administration (when possible) to reduce Treg/MDSC immune suppression and drive antitumor effector responses where they count; in the tumor milieu.

## 12. Expanding Effector Cell Types in Tumors

Relying on CTLs to eradicate tumors may be not always be effective, even when they have sufficient CD4 “help,” particularly when faced with tumor cells that have downregulated tumor antigen/s and/or surface MHC class I molecules. Therefore, expanding the types of immune cells that can exert antitumor effector function in the tumor microenvironment to other cells types, in particular, innate cells, may be beneficial to the cancer-bearing host. We have shown that neutrophils (PMN) can collaborate with CTLs to eradicate large tumor burdens [29]. The nature of this collaboration is unclear, but our data showing that PMNs could reduce tumor burden in the absence of T cells implies that they were directly killing tumor cells, or other cells, within the tumor microenvironment. As mentioned above, M1 cells can phagocytose tumor cells and mediate a proinflammatory response. Thus, reprograming M2 TAMs and MDSCs into M1 cells appears to be a rational therapeutic approach. NK cells can also be potent antitumor effector cells and their capacity to lyse MHC class I^low^ cells may be particularly useful when dealing with immunoedited tumor cells in advanced cancer.

## 13. The Important Role of Agonist Anti-CD40 in Modifying the Tumor Microenvironment

Several studies including ours have shown that agonist anti-CD40 Ab is a potent tumor microenvironment modifier. Indeed, in dozens of studies of immunotherapeutic combinations with TLR agonists, cytokines, checkpoint blockers, or chemotherapy, agonistic anti-CD40 Ab has usually proven to be an essential component. Anti-CD40 Ab has a variety of roles—it targets CD40^+^ tumor blood vessels such that they permit T cell traffic [24]; CD40^+^ B cells in tumors, spleen, and lymph nodes (LNs) such that they secrete increased levels of autoantibodies directed against antigen expressed on tumor cells [25]; and CD40^+^ DCs such that they upregulate costimulatory markers and release IL-12 to activate CD8^+^ T cells [88] and stimulate a specific CTL response to cross-presented tumor antigens [89, 90]. CD40 activation also “drives” cross-primed T cells out of the draining lymph node into the systemic circulation [91].

When anti-CD40 Ab was combined with other immune enhancing agents such as local IL-2 [29], local TLR-7 agonists [92], or systemic cytotoxic chemotherapy [93], the generation of a potent antitumor CD8^+^ cytotoxic T lymphocyte (CTL) response was seen. Indeed, local administration of TLR-3, TLR-7, and TLR-9 agonists mediates tumour rejection by “reactivating” preexisting intratumoural CD8 T cells in a Type I IFN dependent manner [94, 95]. All of these therapeutic approaches induced a local and systemic immune response resulting in increased tumor cell death via cytotoxicity or via activated antigen presenting cells leading to increased tumor antigen delivery to the local cross-presentation pathway in draining LNs.

Combining anti-CD40 mAb with IL-2 promoted and maintained a long-term protective memory [31]. Whilst the primary response was attributed to infiltrating tumor-antigen specific CD8^+^ T cells and PMNs [29], we found that NKs cells played a key role in the generation and maintenance of memory T cell responses [32]. Another group found similar success with the IL-2/anti-CD40 mAb combination in an advanced, metastatic model of renal carcinoma; the antitumor effect was dependent on CD8^+^ T cells and proinflammatory cytokines. They showed significant increases in DC and T cell numbers in treated mice, implying CD40-CD40L interactions and the induction of a lasting antitumor response [96, 97]. The induction of IFN-*γ* was found to reduce the tumor immunosuppressive environment by decreasing the number of MDSCs, Tregs, and Th2 cytokines whilst increasing Th1 cytokine levels and recruiting an inflammatory infiltrate into the tumor site. This study further confirmed the requirement for the combination of IL-2 or IL-15 with anti-CD40 mAb for tumor regression versus the partial effects seen with anti-CD40 mAb treatment alone in large tumor burdens [29]. Combination with IL-15, a potent activator of NK and T cells, has also proved beneficial in a murine model of colon cancer [98]. Finally, we have recently shown that the IL-2/anti-CD40 Ab combination reprograms M2 macrophages to M1 macrophages [43].

## 14. Methods Used to Target the Tumor Microenvironment in Human Studies

Local intratumoral immunotherapy in animals and humans has been delivered by a variety of approaches, including cytokines, gene therapy vectors, sustained release gels, pumps, nanoparticles, and targeted systemic therapy that produces local effects. A large number of human and animal studies have shown that direct intratumoral injection of cytokines, antibodies, cytotoxic chemotherapy, and so forth is safe, feasible, and effective. Direct injection has been performed on several human tumors under, for example, computed tomographic guidance. In humans, intratumoral injections have been successfully performed in melanoma [99], liver cancer [100], lung cancer [101–103], colorectal cancer [104], pancreatic cancer [105], cervical chordoma [106], Wilms' tumor and neuroblastoma [107], cystic craniopharyngioma [108], head and neck tumors [109], glioblastoma multiforme [110], and even in difficult to access tumors such as mesothelioma [111] that begin as discrete plaques and nodules and develops into a sheet-like neoplasm lining the pleural cavity [112].

**Figure 3:**
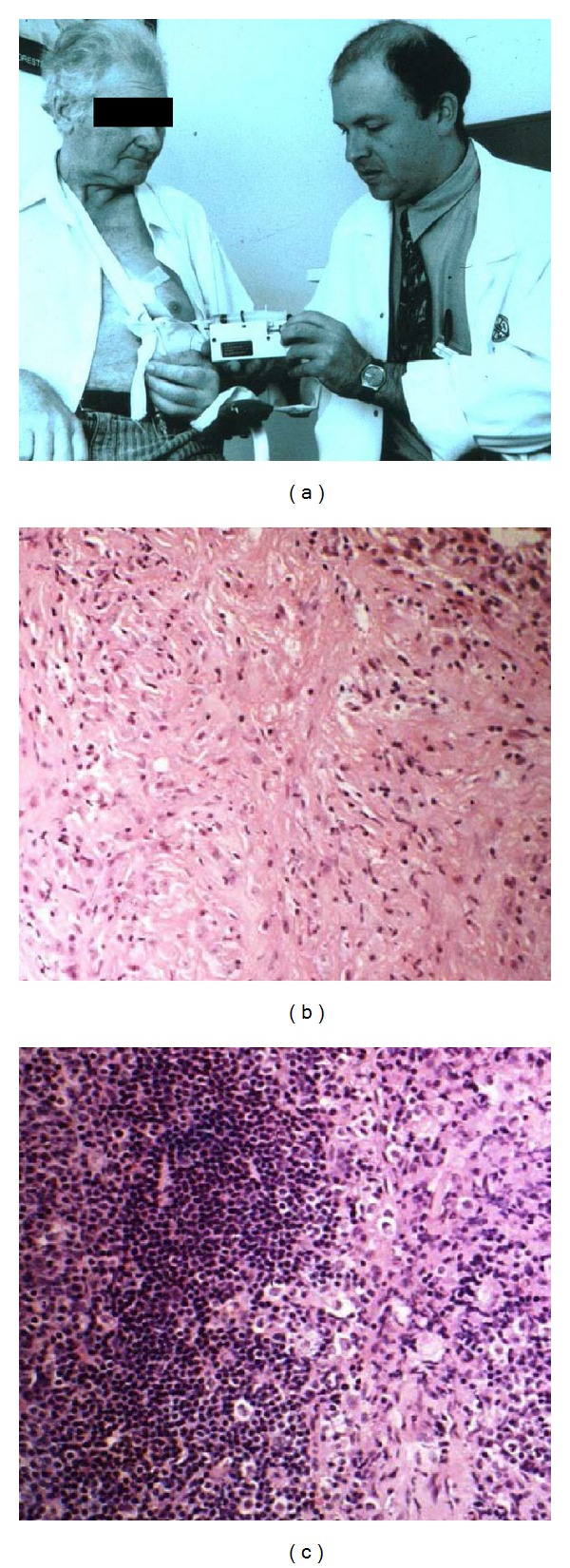
Induction of local and widespread antitumor reactivity with continuous cytokine infusion into tumors. Recombinant human GM-CSF was administered continuously into the tumor of a patient with pleural mesothelioma using a pump attached to an intratumoral cathers via a subcutaneous port (a) Tumor biopsies (H&E) taken before (b) and after (c) 8 weeks of continuous intratumoral infusion of GM-CSF. Shown is a marked influx of immune cells into uninfused tumor following the therapy. This patient exhibited a generalized partial response (>50% diffuse tumor reduction).

We have studied the effects of continuous cytokine infusion into human tumors. Using a catheter with multiple sideports inserted into tumors under CT guidance, linked to a subcutaneous port and followed by 8 weeks of continuous cytokine infusion (GMCSF or IFN alpha), we were able to demonstrate local and distal tumor shrinkage with few systemic side effects [111, 113]. Importantly, some clinical responses were seen and an intense local and distal immune infiltrate was observed in responders (Figure 3). Nonetheless, this sort of direct injection or infusion is difficult and not always feasible in human cancers because of anatomical location and sepsis issues. As a result, more sophisticated and technologically advanced approaches have been tested. Tumor endothelia have been targeted by genetically engineering agonistic CD40 antibodies on peptides that specifically bind tumor vasculature and not normal vessels in transgenic mouse models [24]. This proof-of-principle method allows for tumor targeting via intravenous injection and avoids systemic toxicity.

Bispecific and trispecific antibodies are a new technology consisting of binding sites for two or three different antigens that can be used to target, for example, CD40^+^ endothelial and immune cells or antigen-specific tumor cells in the tumor microenvironment. This can be achieved by construction of diabodies that target a tumor antigen on one site and another molecule such as CD40 or IL-2 on another binding site [39]. These antibodies can also be used to bridge two different cell types together, for example, DCs and T cells. Bispecific diabodies that simultaneously target human CD40 and CD28 on naïve T cells have been constructed [39].

Nanoparticles are a relatively new and promising mode of targeting tumors and several different types are being developed including liposomes, polymeric micelles, dendrimers, superparamagnetic iron oxide crystals, and colloidal gold [114, 115]. Some strategies are based on the enhanced permeability and retention effect of the tumor vasculature [114]. Macromolecules with a molecular weight range of 15,000–70,000 g/mol can accumulate in solid tumors with larger molecules being retained longer. Size may also prevent diffusion back into the circulation. Importantly, whilst liposomes may extravasate from leaky tumor vessels, they do not diffuse away from the tumor even after a week [115] and they can transport several molecules into the tumor microenvironment. In contrast, others rely on specificity via ligand-directed binding of nanoparticles to receptors expressed by cells in the tumor microenvironment [114] and again can deliver molecules into tumors.

Gene therapy is another tumor targeting strategy that aims to deliver therapeutic genes into tumors, whereupon their expression in transfected tumor and/or stromal cells is intended to alter the course of disease. For example, we have shown that local expression of transgenes can enhance sensitivity of tumor cells to therapy [116–120], modulate local and antitumor immune responses, block angiogenesis, or normalize tumor vasculature. We conducted immunogene therapy in six mesothelioma patients aiming to boost antitumor immune responses by injecting tumor lesions with a recombinant vaccinia virus expressing human IL-2, VVIL2 [121]. No systemic or local toxicities were observed and, despite antibody responses, VV-IL-2 mRNA gene expression persisted in tumor biopsies for up to 3 weeks, albeit at low levels. There was no evidence of excretion of virus or transmission to contacts. A T cell infiltrate was detected in 50% of tumor biopsies at the site of injection. However, no clinical responses were observed, suggesting that the amount of IL2 produced was limited and thus this treatment approach needs further development [122]. It is not yet clear which cells in the tumor microenvironment were infected by the VV vector. However, our in vitro studies showed that mesothelioma tumor cells were highly permissive to infection by VVcytokine vectors resulting in significant cytokine production and impaired proliferation, whilst macrophages secreted low levels of IL-2 suggesting resistance to overt infection [123]. We have also shown that human dendritic cells are readily infected with the VVIL-2 vector and that that they become efficient antigen presenting cells that secrete high levels of the immunostimulatory cytokine IL-12 [124]. Our murine proof-of-principle in vivo studies of murine mesotheliomas showed that i.t. injection of the parent VV could not hinder tumor progression. In contrast, the VV-IL-2 constructs induced profound tumor regression [123]. This local effect may provide activation and survival signals to infiltrating tumor antigen-specific T cells and modulate tumor blood vessels, as we have shown in mouse models [125, 126].

Others have used DNA vaccines directed against targets overexpressed on tumor cells or on stromal cells in proof-of-principle studies showing elimination of primary and metastatic tumors. One study examined whether tumor targeted delivery of a synthetic STAT-3 inhibitor using a ligand-targeted nanoparticle combined with an HER-2 DNA vaccine could improve immunity against HER-2^+^ breast cancer [127]. The researchers reported increased IFN-*γ*, p-STAT-1, GM-CSF, IL-2, IL-15, and IL-12b and reduced TGF-*β*, IL-6, and IL-10 protein expression in tumors in association with infiltrating activated CD8^+^ T cells, M1 macrophages, and DCs. These changes correlated with delayed growth of orthotopic breast tumors and prevented tumor recurrence.

## 15. Future Prospects

**Figure 4:**
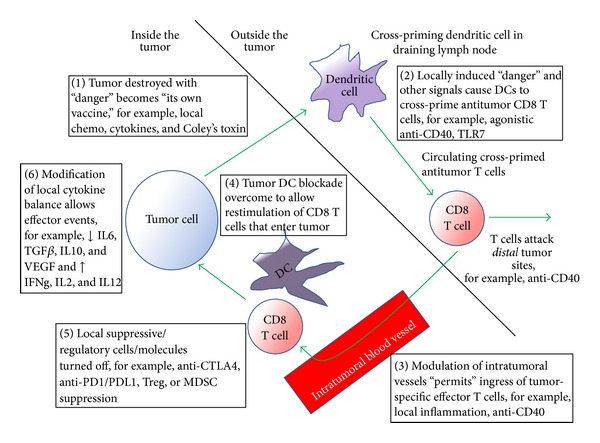
Illustration of the ways in which “Trojan Horse” approaches immunotherapy might be effective. Tumors that are destroyed by local therapy (1) deliver tumor antigens into the draining lymph node (2), that is, the tumor acting as its own vaccine. This cross-primes an antitumor T cell response which, when the tumor vasculature is modified to encourage entry into the tumor (3), results in the accumulation of effector T cells in the tumor. Once local suppressive defenses are overcome (4) and a milieu that is conducive to local effector events is established (5), tumor destruction can result.

There is convincing evidence that local immunotherapy approaches can induce tumor regression locally and systemically via a variety of pathways, such as induction of immunogenic tumor cell death, modification of the local cytokine, and regulatory cell milieu to reverse suppression, generation of an immune-permissive milieu, and direct stimulation or restimulation of antitumor effector responses (summarized in Figure 4 and Table 1). These represent strong rationales for further studies using this “Trojan Horse” approach.

Notwithstanding the technical difficulties associated with direct intratumoral immunotherapy, the logic of the approach, combined with strong preclinical data, suggests that further studies in animals and humans are warranted. For example, agonistic anti-CD40 Ab has proven to be a very effective local immunotherapy. Agonistic anti-CD40 Ab is currently being used systemically in clinical trials with promising results although there are some toxicity issues. Study using locally administered anti-CD40 Ab in cancer patients that might avoid toxicity and generate an even better response seems warranted.

Overall, improved delivery methods that enable sustained infusion of agents are what is needed. Intratumoral gene therapy was intended to achieve that goal but weak transgene expression and immunogenicity issues have made that approach nonfeasible [113]—they were unable to deliver the sorts of cytokine levels observed in diseased organs [128]. It will also be vital to select patients for this sort of therapy based on the status of the immune processes within tumors. For example, if lack of immunogenicity is the problem, then increasing antigen load, costimulation, or modification of cytokine environments would be considered, depending upon which of those is limiting. If local immunosuppression is also present, then checkpoint blockade or other approaches will be required.

## Figures and Tables

**Table 1:** Immunotherapies with a demonstrated local antitumour effect.

Agent	Mechanism	Reference
IL-2/anti-CD40 mAb	Licensing of local and systemic CTL	[24, 25, 29, 40, 91]
Anti-CD40 mAb	Licensing of CTL by dendritic cells but can also promote angiogenesis	[24, 27, 88–91]
Anti-CTLA-4 mAb	Immunes checkpoint blockade, enhances Teff response by blocking CTLA-4 inhibitory signals, and limits Treg function	[62–66]
Anti-PD-1 mAb	Blocks PD-1 mediated T cell inhibition, particularly when used in conjunction with anti-CTLA4 therapy	[68–73]
p300	Targeted inhibition of p300 impairs Treg function	[56]
TLR7 agonist	Activates DCs	[92]
TLR 3/9 agonists	TLR mediated, Type I IFN dependent activation of preexisting intratumoural CD8 T cells	[94, 95]
Viral-cytokine constructs	Infection of different cell types in tumour microenvironment, persistent cytokine expression and increased antigen cross-presentation	[111, 113, 121–124]
Cytokine or alloMHC transfection	IL-2, IL-12, GMCSF, and B7.1 enhance antitumour immunity	[116–120, 125]
Cyclophosphamide	Potentiation of antitumour immunity by targeting of Treg	[58–60]
Chemotherapy	Direct tumour cytotoxicity	[99–110]
